# 1,2-Bis[2-(2-nitro-1*H*-imidazol-1-yl)eth­oxy]ethane

**DOI:** 10.1107/S1600536808023726

**Published:** 2008-08-06

**Authors:** Shu-Xian Li, Lin Zhu, Hua-Min Li, Hoong-Kun Fun, Suchada Chantrapromma

**Affiliations:** a Department of Chemistry, Beijing Normal University, Beijing 100875, People’s Republic of China; b X-ray Crystallography Unit, School of Physics, Universiti Sains Malaysia, 11800 USM, Penang, Malaysia; c Crystal Materials Research Unit, Department of Chemistry, Faculty of Science, Prince of Songkla University, Hat-Yai, Songkhla 90112, Thailand

## Abstract

In the crystal structure, the title compound, C_12_H_16_N_6_O_6_, lies on an inversion centre. The mol­ecule has an anti­periplanar conformation with respect to the C—C bond of the central ethane unit and the two imidazole rings are parallel to each other. The dihedral angle between the imidazole ring and the mean plane of the C and O atoms of the bis­(eth­oxy)ethane group is 76.04 (6)°. The mol­ecules are stacked along the *c* axis through a weak C—H⋯O inter­action and a π⋯π inter­action between the imidazole rings with a centroid–centroid distance of 3.5162 (6) Å. An intramolecular C—H⋯O hydrogen bond is also present.

## Related literature

For bond-length data, see: Allen *et al.* (1987 ▶). For the applications of nitro­imidazoles, see, for example: Abdel-Jalil *et al.* (2006 ▶); Kennedy *et al.* (2006 ▶); Nagasawa *et al.* (2006 ▶); Nunn *et al.* (1995 ▶).
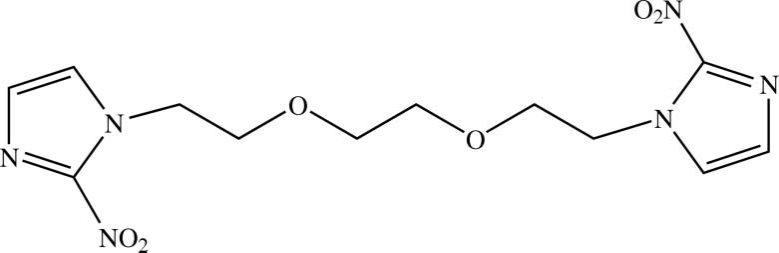


## Experimental

### 

#### Crystal data

C_12_H_16_N_6_O_6_
*M* *_r_* = 340.31
Monoclinic, 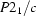

*a* = 7.0534 (1) Å
*b* = 15.5792 (2) Å
*c* = 6.8069 (1) Å
β = 99.560 (1)°
*V* = 737.60 (2) Å^3^
*Z* = 2
Mo *K*α radiation
μ = 0.13 mm^−1^
*T* = 100.0 (1) K
0.40 × 0.30 × 0.15 mm

#### Data collection

Bruker SMART APEXII CCD area-detector diffractometer
Absorption correction: multi-scan (**SADABS**; Bruker, 2005 ▶) *T*_min_ = 0.952, *T*_max_ = 0.982
11084 measured reflections
2140 independent reflections
1864 reflections with *I* > 2σ(*I*)
*R*_int_ = 0.024

#### Refinement

*R*[*F* ^2^ > 2σ(*F* ^2^)] = 0.037
*wR*(*F* ^2^) = 0.106
*S* = 1.05
2140 reflections
141 parameters
All H-atom parameters refined
Δρ_max_ = 0.49 e Å^−3^
Δρ_min_ = −0.32 e Å^−3^

### 

Data collection: *APEX2* (Bruker, 2005 ▶); cell refinement: *APEX2*; data reduction: *SAINT* (Bruker, 2005 ▶); program(s) used to solve structure: *SHELXTL* (Sheldrick, 2008 ▶); program(s) used to refine structure: *SHELXTL*; molecular graphics: *SHELXTL*; software used to prepare material for publication: *SHELXTL* and *PLATON* (Spek, 2003 ▶).

## Supplementary Material

Crystal structure: contains datablocks global, I. DOI: 10.1107/S1600536808023726/is2317sup1.cif

Structure factors: contains datablocks I. DOI: 10.1107/S1600536808023726/is2317Isup2.hkl

Additional supplementary materials:  crystallographic information; 3D view; checkCIF report

## Figures and Tables

**Table 1:** Hydrogen-bond geometry (Å, °)

*D*—H⋯*A*	*D*—H	H⋯*A*	*D*⋯*A*	*D*—H⋯*A*
C4—H4*A*⋯O3^i^	0.977 (13)	2.404 (13)	3.3707 (11)	170.1 (10)
C4—H4*B*⋯O2	0.944 (14)	2.408 (13)	2.8169 (13)	105.9 (9)

Symmetry code: (i) 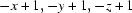.

**Fig. 1.**
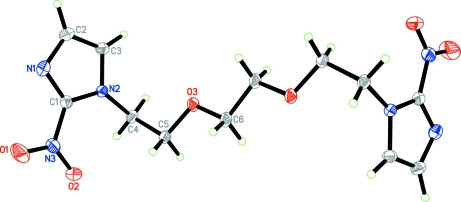
The molecular structure of (I), showing 50% probability displacement ellipsoids and the atomic numbering.

**Fig. 2.**
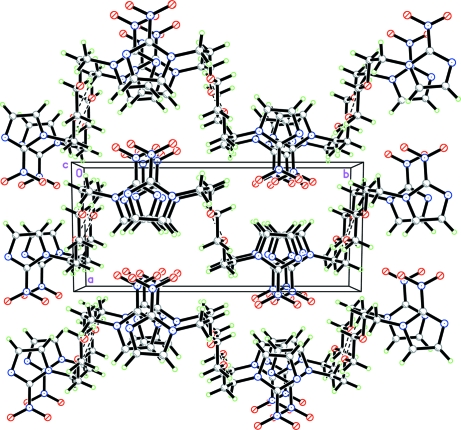
The crystal packing of (I), viewed down the c axis, showing stacking of molecules along the c axis. Hydrogen bonds are shown as dashed lines.

**Fig. 3.**
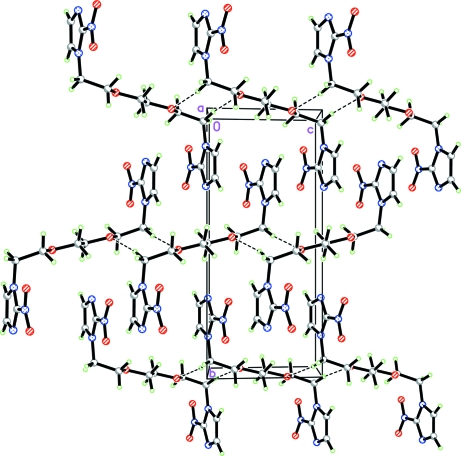
The crystal packing of (I), viewed down the a axis. Hydrogen bonds are shown as dashed lines.

## supplementary crystallographic information

### Comment

Depending on the availability of oxygen in tissue, nitroimidazoles can undergo different intracellular metabolism. In a normal cell, the molecule undergoes reduction to become a potentially reactive species and can be reoxidized in the presence of normal oxygen levels.  In hypoxic tissue, however, the low oxygen concentration is not able to effectively reoxidize the molecule which results in more reactive intermediates that bind with components of hypoxic tissues (Nunn *et al.*, 1995). Thus these compounds can function as hypoxia markers for imaging of hypoxic cells and have received much attention in medicinal and clinic studies (Abdel-Jalil *et al.*, 2006; Kennedy *et al.*, 2006; Nagasawa *et al.*, 2006). In an attempt to develop new hypoxic cell radiosensitizers, we present herein the synthesis and crystal structure of the title nitorimidazole compound, (I).

The molecule of the title compound, C_12_H_16_N_6_O_6_, lies on an crystallographic inversion centre, so the asymmetric unit contains half of the molecule. The molecular structure has an antiperiplanar conformation with the two imidazole rings parallel to each other. The imidazole ring is planar, within a deviation of ±0.003 Å. The nitro group is twisted from the mean plane of imidazole ring with torsion angles O1–N3–C1–N1 = -6.49 (16)° and O2–N3–C1–N1 = 173.61 (9)°. Atoms C5, O3, C6, C5^i^, O3^i^ and C6^i^ [symmetry code: (i) 1 - *x*, 1 - *y*, -*z*] lie on the same plane. The interplanar angle between the C5/O3/C6/C5^i^/O3^i^/C6^i^ plane and the imidazole ring (N1/N2/C1–C3) is 76.04 (6)°. The conformation of the ethoxyethane group is (-)-*syn*-clinal with respect to the imidazole ring, which is reflected by the torsion angle N2–C4–C5–O3 = -72.25 (9)°. Bond distances and angles have normal values (Allen *et al.*, 1987).

The crystal packing of (I) in Fig. 2 shows that the molecules are linked by weak C—H···O interactions (Table 1) and stacked into columns along the *c* axis. The molecules in the adjacent columns are in a face-to-face fashion (Fig. 3). The crystal is stabilized by a weak C—H···O interaction (Table 1). A π···π interaction was also observed in the crystal with the *Cg*_1_···*Cg*_1_^ii^ [symmetry code: (ii) *x*, 1/2 - *y*, -1/2 + *z*] distance of 3.5162 (6) Å; *Cg*_1_ is the centroid of the N1/N2/C1–C3 ring.

### Experimental

To a solution of the triethyleneglycol ditosylate (0.458 g, 1.0 mmol) and triethyamine (244 mg, 2.4 mmol) in DMF (10 ml) was added a solution of 2-nitroimidazole (249 mg, 2.2 mmol) in DMF (10 ml) under argon. The mixture was stirred at 313 K for 4 days. After concentration on the rotary unit under reduced pressure, ethyl acetate (80 ml) was then added to the reaction residue, washed with water (20 ml × 3), dried (Na_2_SO_4_) and the organic layer was evaporated to dryness and subjected to chromatography on silica with 50% EtOAc-hexane to afford the desired compound (I) (0.255 g, yield 75%). Analysis calcd for C_12_H_16_N_6_O_6_: C 42.35, H 4.74, N 24.70%; found: C 42.01, H 4.71, N 24.43%. Single crystals suitable for X-ray diffraction analysis were obtained by the slow diffusion of hexane into the dichloromethane solution of the title compound.

### Refinement

All H atoms were located in a difference map and refined isotropically.

### Crystal data

**Table:**

C_12_H_16_N_6_O_6_	*F*_000_ = 356
*M**_r_* = 340.31	*D*_x_ = 1.532 Mg m^−^^3^
Monoclinic, *P*2_1_/*c*	Mo *K*α radiation λ = 0.71073 Å
Hall symbol: -P 2ybc	Cell parameters from 2140 reflections
*a* = 7.0534 (1) Å	θ = 2.6–30.0º
*b* = 15.5792 (2) Å	µ = 0.13 mm^−^^1^
*c* = 6.8069 (1) Å	*T* = 100.0 (1) K
β = 99.560 (1)º	Block, colorless
*V* = 737.597 (18) Å^3^	0.40 × 0.30 × 0.15 mm
*Z* = 2	

### Data collection

**Table:**

Bruker SMART APEXII CCD area-detector diffractometer	2140 independent reflections
Radiation source: fine-focus sealed tube	1864 reflections with *I* > 2σ(*I*)
Monochromator: graphite	*R*_int_ = 0.024
Detector resolution: 8.33 pixels mm^-1^	θ_max_ = 30.0º
*T* = 100.0(1) K	θ_min_ = 2.6º
ω scans	*h* = −9→9
Absorption correction: multi-scan(*SADABS*; Bruker, 2005)	*k* = −19→21
*T*_min_ = 0.952, *T*_max_ = 0.982	*l* = −9→9
11084 measured reflections	

### Refinement

**Table:**

Refinement on *F*^2^	Secondary atom site location: difference Fourier map
Least-squares matrix: full	Hydrogen site location: inferred from neighbouring sites
*R*[*F*^2^ > 2σ(*F*^2^)] = 0.037	All H-atom parameters refined
*wR*(*F*^2^) = 0.106	*w* = 1/[σ^2^(*F*_o_^2^) + (0.0604*P*)^2^ + 0.1626*P*] where *P* = (*F*_o_^2^ + 2*F*_c_^2^)/3
*S* = 1.05	(Δ/σ)_max_ = 0.001
2140 reflections	Δρ_max_ = 0.50 e Å^−^^3^
141 parameters	Δρ_min_ = −0.32 e Å^−^^3^
Primary atom site location: structure-invariant direct methods	Extinction correction: none

### Special details

**Table:**

Experimental. The low-temparture data was collected with the Oxford Cryosystem Cobra low-temperature attachment.
Geometry. All e.s.d.'s (except the e.s.d. in the dihedral angle between two l.s. planes) are estimated using the full covariance matrix. The cell e.s.d.'s are taken into account individually in the estimation of e.s.d.'s in distances, angles and torsion angles; correlations between e.s.d.'s in cell parameters are only used when they are defined by crystal symmetry. An approximate (isotropic) treatment of cell e.s.d.'s is used for estimating e.s.d.'s involving l.s. planes.
Refinement. Refinement of *F*^2^ against ALL reflections. The weighted *R*-factor *wR* and goodness of fit *S* are based on *F*^2^, conventional *R*-factors *R* are based on *F*, with *F* set to zero for negative *F*^2^. The threshold expression of *F*^2^ > σ(*F*^2^) is used only for calculating *R*-factors(gt) *etc*. and is not relevant to the choice of reflections for refinement. *R*-factors based on *F*^2^ are statistically about twice as large as those based on *F*, and *R*- factors based on ALL data will be even larger. The highest residual electron density peak is located at 0.76 Å from C6 and the deepest hole is located at 1.04 Å from C1.

### Fractional atomic coordinates and isotropic or equivalent isotropic displacement parameters (Å^2^)

**Table:**

	*x*	*y*	*z*	*U*_iso_*/*U*_eq_	
O1	−0.11911 (13)	0.21335 (6)	0.31449 (16)	0.0405 (2)	
O2	−0.12171 (11)	0.35244 (5)	0.34055 (13)	0.0319 (2)	
O3	0.39505 (9)	0.47777 (4)	0.21737 (9)	0.01816 (16)	
N1	0.25143 (13)	0.20738 (5)	0.49696 (13)	0.02131 (19)	
N2	0.26946 (11)	0.35116 (5)	0.49565 (11)	0.01551 (17)	
N3	−0.04022 (13)	0.28202 (6)	0.36307 (14)	0.0243 (2)	
C1	0.15885 (13)	0.27986 (6)	0.45100 (14)	0.0183 (2)	
C2	0.43305 (14)	0.23267 (6)	0.57559 (14)	0.0209 (2)	
C3	0.44699 (13)	0.32065 (6)	0.57493 (13)	0.01808 (19)	
C4	0.22148 (14)	0.44265 (6)	0.47476 (13)	0.01791 (19)	
C5	0.20547 (13)	0.47446 (6)	0.26222 (13)	0.01829 (19)	
C6	0.39581 (14)	0.49935 (6)	0.01416 (13)	0.0190 (2)	
H2	0.535 (2)	0.1921 (9)	0.625 (2)	0.024 (3)*	
H3	0.5512 (19)	0.3594 (8)	0.6195 (19)	0.020 (3)*	
H4A	0.3268 (19)	0.4727 (8)	0.5584 (19)	0.020 (3)*	
H4B	0.1055 (19)	0.4504 (8)	0.5245 (19)	0.022 (3)*	
H5A	0.1496 (18)	0.5319 (8)	0.2511 (18)	0.020 (3)*	
H5B	0.1250 (17)	0.4375 (8)	0.1698 (18)	0.017 (3)*	
H6A	0.3216 (18)	0.4569 (8)	−0.0729 (19)	0.021 (3)*	
H6B	0.3398 (17)	0.5550 (8)	−0.0139 (18)	0.017 (3)*	

### Atomic displacement parameters (Å^2^)

**Table:**

	*U*^11^	*U*^22^	*U*^33^	*U*^12^	*U*^13^	*U*^23^
O1	0.0272 (4)	0.0366 (5)	0.0539 (6)	−0.0129 (3)	−0.0041 (4)	−0.0045 (4)
O2	0.0186 (4)	0.0345 (4)	0.0408 (5)	0.0023 (3)	−0.0004 (3)	0.0128 (3)
O3	0.0189 (3)	0.0223 (3)	0.0136 (3)	−0.0025 (2)	0.0038 (2)	0.0027 (2)
N1	0.0249 (4)	0.0174 (4)	0.0216 (4)	0.0000 (3)	0.0037 (3)	0.0023 (3)
N2	0.0152 (3)	0.0162 (4)	0.0152 (3)	0.0008 (3)	0.0028 (3)	0.0021 (2)
N3	0.0187 (4)	0.0287 (5)	0.0246 (4)	−0.0047 (3)	0.0009 (3)	0.0043 (3)
C1	0.0166 (4)	0.0198 (4)	0.0183 (4)	−0.0018 (3)	0.0021 (3)	0.0028 (3)
C2	0.0217 (5)	0.0206 (4)	0.0199 (4)	0.0050 (3)	0.0024 (3)	0.0018 (3)
C3	0.0156 (4)	0.0208 (4)	0.0173 (4)	0.0021 (3)	0.0013 (3)	0.0006 (3)
C4	0.0216 (4)	0.0155 (4)	0.0179 (4)	0.0033 (3)	0.0069 (3)	0.0023 (3)
C5	0.0196 (4)	0.0177 (4)	0.0183 (4)	0.0019 (3)	0.0054 (3)	0.0039 (3)
C6	0.0218 (5)	0.0213 (4)	0.0139 (4)	−0.0030 (3)	0.0035 (3)	0.0035 (3)

### Geometric parameters (Å, °)

**Table:**

O1—N3	1.2258 (12)	C2—H2	0.977 (14)
O2—N3	1.2361 (12)	C3—H3	0.961 (13)
O3—C5	1.4211 (11)	C4—C5	1.5156 (12)
O3—C6	1.4243 (10)	C4—H4A	0.977 (13)
N1—C1	1.3160 (12)	C4—H4B	0.943 (13)
N1—C2	1.3620 (13)	C5—H5A	0.976 (12)
N2—C1	1.3623 (11)	C5—H5B	0.964 (12)
N2—C3	1.3641 (11)	C6—C6^i^	1.5140 (19)
N2—C4	1.4664 (11)	C6—H6A	0.980 (13)
N3—C1	1.4324 (13)	C6—H6B	0.958 (13)
C2—C3	1.3741 (14)		
			
C5—O3—C6	111.88 (7)	N2—C4—H4A	105.6 (7)
C1—N1—C2	104.03 (8)	C5—C4—H4A	109.1 (7)
C1—N2—C3	104.96 (8)	N2—C4—H4B	106.8 (8)
C1—N2—C4	131.03 (8)	C5—C4—H4B	111.7 (8)
C3—N2—C4	123.99 (8)	H4A—C4—H4B	110.5 (11)
O1—N3—O2	124.07 (10)	O3—C5—C4	107.06 (7)
O1—N3—C1	117.50 (9)	O3—C5—H5A	109.4 (7)
O2—N3—C1	118.43 (8)	C4—C5—H5A	109.8 (7)
N1—C1—N2	113.79 (8)	O3—C5—H5B	110.8 (7)
N1—C1—N3	122.19 (8)	C4—C5—H5B	111.7 (7)
N2—C1—N3	124.01 (8)	H5A—C5—H5B	108.1 (11)
N1—C2—C3	110.51 (8)	O3—C6—C6^i^	106.69 (9)
N1—C2—H2	122.7 (8)	O3—C6—H6A	109.9 (7)
C3—C2—H2	126.7 (8)	C6^i^—C6—H6A	111.4 (7)
N2—C3—C2	106.70 (8)	O3—C6—H6B	109.8 (7)
N2—C3—H3	120.7 (8)	C6^i^—C6—H6B	109.8 (7)
C2—C3—H3	132.6 (8)	H6A—C6—H6B	109.2 (11)
N2—C4—C5	112.94 (7)		
			
C2—N1—C1—N2	−0.18 (11)	C1—N1—C2—C3	−0.23 (11)
C2—N1—C1—N3	−179.90 (9)	C1—N2—C3—C2	−0.61 (10)
C3—N2—C1—N1	0.51 (11)	C4—N2—C3—C2	177.86 (8)
C4—N2—C1—N1	−177.81 (8)	N1—C2—C3—N2	0.54 (10)
C3—N2—C1—N3	−179.78 (9)	C1—N2—C4—C5	−77.42 (12)
C4—N2—C1—N3	1.90 (15)	C3—N2—C4—C5	104.54 (10)
O1—N3—C1—N1	−6.49 (16)	C6—O3—C5—C4	174.54 (7)
O2—N3—C1—N1	173.61 (9)	N2—C4—C5—O3	−72.25 (9)
O1—N3—C1—N2	173.82 (9)	C5—O3—C6—C6^i^	−179.94 (9)
O2—N3—C1—N2	−6.08 (15)		

Symmetry codes: (i) −*x*+1, −*y*+1, −*z*.

### Hydrogen-bond geometry (Å, °)

**Table:**

*D*—H···*A*	*D*—H	H···*A*	*D*···*A*	*D*—H···*A*
C4—H4A···O3^ii^	0.977 (13)	2.404 (13)	3.3707 (11)	170.1 (10)
C4—H4B···O2	0.944 (14)	2.408 (13)	2.8169 (13)	105.9 (9)

Symmetry codes: (ii) −*x*+1, −*y*+1, −*z*+1.
